# Proteome-wide remodelling in *Klebsiella pneumoniae* during step-wise adaptive evolution against colistin

**DOI:** 10.1371/journal.pone.0358791

**Published:** 2026-09-24

**Authors:** Sevaram Singh, Lovnish Thakur, Sapna, Shailesh Kumar, Manoj Kumar, Himanshu, Sameer Maitry, Yashwant Kumar, Pramod Kumar Gautam, Niraj Kumar

**Affiliations:** 1 BRIC-Translational Health Science and Technology Institute, NCR Biotech Science Cluster, Faridabad, Haryana, India; 2 Jawaharlal Nehru University, New Delhi, Delhi, India; 3 Department of Biochemistry, AII India Institute of Medical Sciences, New Delhi, Delhi, India; Nitte University, INDIA

## Abstract

Short-duration exposure (SDE) of antibiotics is among the major drivers of the emergence of antimicrobial resistance (AMR) among bacterial pathogens. *Klebsiella pneumoniae* is a critical priority bacterial pathogen and rapidly acquiring MDR/XDR traits even against major antibacterial classes of antibiotics. Since colistin is a last-resort antibiotic for treating drug-resistant *K. pneumoniae* infections and the development of new antibiotics is resource-intensive, understanding how antibiotic-resistance emerges following SDE is crucial for devising strategies to preserve antibiotics for future use. With this aim, we developed colistin (1x, 2x, and 4x MICs)-resistant *K. pneumoniae* using SDE of colistin and protein profiled to understand the emergence of SDE-induced colistin resistance. Interestingly, 1xMIC-adapted cells showed an ~ 8-fold increase in MIC for colistin, with no further rise at 2x or 4x MIC-adapted cells, indicating that the emergence of AMR can be a non-linear adaptive response and SDE of even 1x MIC-dose alone can trigger rapid physiological remodelling and target modification. Proteomic profiling identified 1379 proteins, including 245, 383, and 175 differentially expressed proteins (DEPs) in 1x, 2x, and 4x MIC-adapted cells, respectively. Among these, 75 DEPs were common across all conditions, representing a core proteomic signature consistently altered upon exposure and represented *valS*, *arnA*, *arnB*, *pheT,* and *thrS* the top upregulated proteins, and *gjJ18*, *kphS*, *B5L96*, *potD,* and *phoU* the top downregulated proteins. Functional enrichment highlighted catalytic, antioxidant, and binding activities, whereas protein–protein interaction (PPI) analysis revealed metabolic reprogramming, involving central carbon/amino acid metabolism and transcription/translation machinery associated with reduced antibiotic sensitivity. Lipopolysaccharide (LPS) modification (*arnA*, *arnB*, and *phoU*) and biomolecular synthesis (*valS*, *pheT*, and *thrS*) were the key functions altered during colistin adaptation. Since elevated *arnA*, *arnB* and *phoU* are known to reduce colistin binding to the bacterial surface, they may be facilitating the emergence of antibiotic resistance. Taken together, this research provides insight into SDE–induced colistin resistance in *K. pneumoniae* and may help to identify potential therapeutic and diagnostic interventions.

## Introduction

Antimicrobial Resistance (AMR) has emerged as one of the major escalating threats to global public health since the emergence of resistant bacterial infections limits the therapeutic options, resulting in extended hospital stays and increased healthcare expenses, morbidity and mortality [1–4]. Currently, ~ 0.7 million people die globally each year of antibiotic-resistant bacterial infections, and this is estimated to increase to ~10 million deaths per year by 2050, including 4.1 million deaths from Africa and 4.7 million from Asia, and cost $100 trillion for their treatment and infrastructure development [5–9]. Therefore, understanding the mechanisms regulating the emergence (and/or spread) of AMR among bacterial pathogens is of critical importance.

As per the World Health Organization (WHO) and Department of Biotechnology Government of India(DBT GOI), *ESKAPE* pathogens (*Enterococcus faecium*, *Staphylococcus aureus*, *Klebsiella pneumoniae*, *Acinetobacter baumannii*, *Pseudomonas aeruginosa*, *Enterobacter* species) fall under the most critical and high-priority bacterial pathogens that need urgent attention and are a looming threat to public health [10,11]. Among *ESKAPE, Klebsiella pneumoniae* (*K. pneumoniae*), a gram-negative γ-proteobacteria belonging to the family *Enterobacteriaceae*, is one of the critical priority bacterial pathogen that causes various life-threatening infections, including hospital-acquired infections (such as pneumonia, bloodstream infections, urinary tract infection) and community-acquired infections (such as necrotizing pneumonia, pyogenic liver abscesses and endogenous endophthalmitis), shows high prevalence and rapidly acquiring multidrug resistance (MDR), extensively-drug resistance (XDR) phenotype to major antibacterial classes (such as cephalosporins, aminoglycosides, fluoroquinolones, and carbapenems) [6,12,13]. Carbapenems are among the primary antibiotics used to treat *K. pneumoniae* infections, however, the increasing global prevalence of carbapenem resistance is severely limiting treatment options for MDR/XDR strains [14]. As a consequence, colistin (polymyxin E) has re-emerged as one of the few available last-resort treatments for carbapenem-resistant *K. pneumoniae* (CRKP) infections [15]. Colistin targets the lipid A component of lipopolysaccharide (LPS) on the outer membrane of Gram-negative bacteria and disrupts their bacterial membrane, leading to bacterial cell death [16]. However, the increasing prevalence of colistin resistance globally, including in the Indian clinical settings, poses a significant threat to the continued effectiveness of colistin as a last-resort antibiotic [17–19]. And, if the bacteria develop resistance against both (carbapenem and colistin), there won’t be many options left for treating such illnesses.

Recent advancements in proteomics have significantly enriched knowledge of the molecular mechanisms of colistin resistance *K. pneumoniae*. Genomic, transcriptomic, proteomic and metabolomic -based studies have highlighted the membrane remodelling, lipid A modification and oxidative stress responses as the mechanism to develop resistance upon colistin exposure [20–28]. These investigations primarily investigated only the endpoint (resistant) phenotype with respect to the control (sensitive). The molecular events that occur during the progressive evolution of resistance remained unexplored and hence, how and when this resistance emerges during the process of emergence of AMR remained unknown, limiting the development of novel strategies to tackle AMR.

One of the major cause of the emergence of AMR is the inappropriate use (abuse, misuse, and overuse) of antibiotics [29]. Short-duration exposure (SDE; *i.e.,* treatment plans are cut short or ended too soon) of antibiotics often results in incomplete/partial eradication of the pathogen, even when the antibiotics are administered at therapeutically appropriate doses. The left-over survivors may be the selected population of resistant and/or phenotypically adapted cells, which may have an increased minimum inhibitory concentration (MIC) [30]. This is primarily because exposure to the antibiotic, though for an inadequate duration, creates an evolutionary pressure on the pathogen to induce genetic modifications and may induce alterations in the gene sequences or shifts in protein expression patterns. However, despite its significant contribution to the emergence of AMR, the molecular mechanism of SDE-induced resistance in bacterial pathogens remains unknown.

Hence, in this investigation, the bacterial cells were exposed to colistin with MIC followed by supra-MIC (≥MIC; 2x and 4x MIC) sequentially with the aim of killing the majority (~90%) of the bacterial population, and left-over survivors were allowed to grow until comparable growth to control/wild type (WT) cells is achieved. This recurrent selection process provided a hetero-resistance cell model (*i.e.*, coexistence of subpopulations that may vary in their levels of antibiotic susceptibility (≥ the concentration of antibiotics used) within an otherwise genetically identical (isogenic) bacterial population) that may enable investigation of proteome-level cellular changes to reveal coordinated regulation across the several functional modules (cellular biomolecular transport systems, metabolic pathways, and/or cell envelope architecture etc.) for bacterial survival and adaptation. The investigation of sequential bacterial adaptive responses under antibiotic selective pressure enabled the characterisation of both, stage-specific proteomic response as well as conserved adaptive signature, throughout the emergence of resistance, irrespective of the doses. This knowledge could lead to the discovery of new therapeutic and diagnostic targets that may help in tackling SDE-induced antibiotic resistance.

## Materials and methods

### Bacterial strains, media, and antibiotics

The Institutional Biosafety Committee of Translational Health Science and Technology Institute (THSTI), Faridabad gave its approval for this study (THS/226/2020). For research purpose, archived clinical bacterial isolate was retrieved in Jan 2021 from THSTI. During or after data collection, the authors did not have access to information that would have allowed them to identify specific participants. The study was a part of a project approved by Indian Council of Medical Research under the title “Identification of early biomarker for emergence of antimicrobial resistance among bacteria due to inadequate dose and duration (IBSC Reference number #226/2020)”.

Carbapenem resistant bloodstream clinical isolate of *K. pneumoniae* was precultured aerobically in Muller Hinton Broth/Agar (MHB/MHA) (Difco™) overnight at 37°C and 250 rpm followed by a fresh inoculation in MHB (1: 500 v/v) until the mid-exponential phase of growth [O.D. at 630nm = 0.4–0.6, ~ 4 hrs. post-inoculation] was achieved [31]. Freshly aliquoted cells were cultured with/without selection pressure for laboratory-based evolution of antibiotic resistance. For pellet preparation, cultures equivalent to 10^6^ cells/mL were centrifuged for 10 min at 4°C and 8000 g. The supernatant was discarded and the cell pellets were washed with 4°C cold phosphate buffered saline (PBS) and then stored at −80°C till further analysis. Stock solution of colistin (Sigma Cat. No. C1511-10MU) was prepared in autoclaved milli-Q water stored at −20°C till one week or used.

### Minimum inhibitory concentration (MIC) evaluation via Broth microdilution (BMD) and E-strip method

MIC of the bacterial strains against colistin was established using 34^th^ M100 Clinical and Laboratory Standards Institute (CLSI) 2024 guidelines for broth microdilution (BMD) [32]. In short, bacterial culture was inoculated into MHB at 37°C and 250 rpm for 12–16 hrs. Overnight culture was diluted in 1:1000 (v/v) in fresh 5mL MHB and incubated at 37°C and 250 rpm for 2–2.5 hrs. to get the exponential phase culture (absorbance ~0.4–0.6). Thereafter, the 96-well round bottom plates (Coring® Cat No:3799) were seeded with 10^6^ cells/mL (optical density (O.D.)-based cell number measurement) and with antibiotics as per the CLSI guidelines. The cell growth (O.D.) was measured using a microplate absorbance spectrophotometer at 630nm (BIO-RAD, xMark^TM^ Microplate absorbance Spectrophotometer). Secondly, for MIC evaluation using the E-strip method, bacterial lawn was created on MHA plates using bacterial culture with an absorbance ~0.4–0.6 and sterile cotton swab (Hi-media). Sterile E-strips (Ezy MIC™ Strips-Hi-media) containing the gradient antibiotics of colistin were placed on the agar plate bacterial lawn. Plates were incubated at 37°C for 12–16 hrs. and the zone of inhibition (mm) was measured to calculate MIC and recorded as images using ChemiDoc™ MP Imaging System (Bio-Rad).

### Colistin resistant *K. pneumoniae* model establishment and validation

After establishing the MIC of the colistin using BMD method, a fixed inoculum of *K. pneumoniae* cells were exposed to MIC concentration (1x MIC) of colistin antibiotics and incubated for 4 hrs. and then the absorbance was taken at 630nm. Later, a small fraction of incubated sample was plated on MHA plate and incubated overnight. Next day, few discrete CFUs grew on agar plate were picked and expanded by resuspend into broth without any selection pressure (antibiotic) and incubate in shaker incubator at 37°C and 250 rpm for 8–10 hrs. The cells were sub-cultured and maintained for next 3 days to de-stress the cells. Further, the pathogen was treated again with the MIC of colistin, incubated for 4 hrs. and the absorbance was taken at 630nm. The sample was plated and discrete CFUs were expanded and maintained for next 3 days. The process was repeated until the growth rate of survivors becomes comparable to the WT/control [O.D.: ≥ 0.6 (in ≥3 consecutive biological replicates)]. Once the *K. pneumoniae* cells achieved comparable growth in the presence of antibiotics similar to control/WT after 4 hrs. of incubation, the cells were considered adapted and stocked (glycerol stock). To achieve this sequential adaptation process, bacterial populations require 5 times exposure-recovery cycles over about 8 days to adapt to 1x MIC, 4 times exposure-recovery cycles over 7 days to adapt to 2x MIC, and 4 times exposure-recovery cycles over 7 days to adapt to 4x MIC. For growth validation, adapted cells (~10^6^ cells/mL) were grown with/without antibiotics for 4 hrs. followed by growth evaluation using absorbance and plating on MHA plates. For antibiotic resistance/susceptibility validation, lawn of adapted cells was obtained on MHA plate and E-strips (Ezy MIC™ Strips-Hi-media) was placed, zone of inhibition was measured (to calculate MIC) after overnight incubation at 37°C.

### Pathogen Identification and antimicrobial susceptibility test (AST) characterization

A single colony from adapted bacterial samples grown on MHA were picked and checked for species level identification using Matrix-Assisted Laser Desorption IonizationTime-of-Flight (MALDI-TOF, bioMerieux serial number 51224). Briefly, bacterial colony was smeared on to a machine reading plate followed by adding HCCA (α-cyano-4-hydroxycinnamic acid) matrix solution (1 µL) along with 50% acetonitrile and 2.5% trifluoracetic acid, air dried and analysed using MALDI-TOF. Simultaneously, these samples were subjected to antibiotics susceptibility testing (AST) by VITEK-2 compact system (bioMerieux serial number VK2C17824) using AST Card No.407 (S Tables 1 and 2 in S1 File) according to European Committee on Antimicrobial Susceptibility Testing (EUCAST) guidelines. For this, a single isolated colony was used to form bacterial suspension of 0.5 McFarland standard and suspension was inoculated in VITEK 2 AST card, incubated at 37°C for 12–16 hrs. AST results were obtained in document form and interpreted based on EUCAST guidelines and used for comparative analysis.

### Proteomics

Model *K. pneumoniae* cells representing parent strain 0x (WT), resistant at 1x MIC, 2x MIC and 4x MIC for colistin were cultured by adding respective adapted antibiotics concentration and harvested in the mid-exponential phase of culture [O.D.630nm = 0.4–0.6, ~ 4 hrs. post-inoculation]. Cold PBS was used to wash the cells twice, and the pellet were stored at −80°C until used. 50µg protein was denatured by 8M urea at 1 mg/mL concentration. 10mM DTT was used for reduction for up to 1 hour at 37°C followed by alkylation with 20mM IAA in dark. 50mM ammonium bicarbonate was added to the sample mixture to dilute the urea concentration to 2M. Proteins were digested with trypsin at 1:20 for overnight at 37°C. Following digestion, the resulting peptide mixture was then acidified using formic acid, desalted, and vacuum concentrated. 3 biological replicates for each adapted sample were generated.

### LC-MS/MS conditions

LC-MS/MS was done using a Vanquish™ Neo UHPLC System, connected on-line with a Orbitrap Exploris™ 480 Mass Spectrometer (Thermo Scientific). The trypsin digested samples were desalted offline by C18 column prior to loading them on a Trap (PepMap™ Neo Trap Cartridge 100 A, 5 μm C18 0.3 mm x 5 mm) and analytical nano column (Easy-Spray™ PepMap™ Neo 2 μm C18 75 μm x 150 mm). The mobile phase for HPLC was as follow: water/formic acid (100/0.1%) and acetonitrile/formic acid (80/0.1%). Sample (500 ng) was injected into the column with a flow rate of 300 nl/min. Analytical separation was established by the following gradient conditions: initial 6% B for 2 min, followed by a linear gradient to 31% B in 58 min, followed by another linear gradient to 44% B in 30 min. Following the peptide elution window, the gradient was increased to 100% B in 1 min and held for 9 min.

Data-dependent acquisition experiment was performed on a Orbitrap Exploris™ 480 system having a FAIMS Pro™ Interface. FlexMix calibration solution (Pierce) was used to tune the instrument before data acquisition. Each biological replicate was run once (1 technical replicates) and data was acquired using a nano-spray ionization ion source with a voltage 2000 V, ion transfer tube temperature 280°C, FAIMS voltage −50°C, RF lens 40%, automatic gain control 300%, maximum injection time in auto mode and 1 microscan. The MS was operated at a resolution of 60,000 FWHM and a mass range of m/z 350–1200. A total of 30 survey scans were acquired at 15,000 resolution and 28% HCD collision energy, if exceeding an intensity threshold of 5.0e3 with a 2+ to 6 + charge states. Dynamic exclusion duration was set for 45s.

### Database search for protein identification

Identification of proteins in the samples were performed via database searching against the *K. pneumoniae*, downloaded from the UniProt database (taxon ID 573 and accessed on 13 November 2023) with Proteome discoverer software having CHIMERYS algorithm and INFERYS rescoring. The search parameters were as follows: enzyme trypsin; maximum missed cleavage 2; mass tolerance 10 ppm; static modification Cys carbamidomethyl; dynamic modification Met oxidation; target/decoy strategy using percolator; target FDR 1%. A total of 1,370 proteins were identified and quantitatively examined in all experimental groups (WT, 1x, 2x, and 4x MIC). These proteins comprised both condition-specific proteins that were only found in certain stages of adaptation and proteins that were frequently found in some or all conditions.

### Statistical analyses

The list of identified and normalized proteins in all samples were analysed using Metaboanalyst v.6 Statistical threshold of p-value ≤ 0.05 was applied to determine significance and log2FC was used for identifying upregulated and downregulated proteins in comparison to control/WT. A log2 fold change (log2FC) threshold of ≥ + 1 for upregulated proteins and ≤ −1 for downregulated proteins (corresponding to at least a 2-fold change). All possible comparison groups were created to find the significant proteins in each condition (S Table 3 in S1 File). The list of significant proteins in different conditions was statistically and functionally analysed using volcano, principal component analysis (PCA) and UpSet plots.

### Differentially expressed proteins (DEPs) heatmap and protein-protein interaction (PPI)

Differentially expressed proteins (DEPs) abundance was used to plot heatmap using GraphPad Prism v10.1.0 (GraphPad Software, San Diego, CA, USA) Software Inc. Protein-protein intraction (PPI) network was generated using STRING database (Version:12.0). Interactions were selected based on text mining, experiments, databases, co-expression, neighboured hood, gene fusion, and co-occurrence. The network was visualized using Cytoscape software (Version:3.10.2).

### Quantitative real-time PCR (qRT-PCR)

Mid-log phase *K. pneumoniae* cultures were used to extract total RNA (O.D. 630nm = 0.5–0.6) using RNA Purification Kit (MACHEREY-NAGEL, Ref. No. 740955.250). A NanoDrop spectrophotometer was used to measure the concentration and purity of RNA (A260/280 ≥ 1.8, A260/230 ≥ 2.0).

### Primer design and validation

Gene-specific primers for *arnA*, *arnB*, and *kphS*, and reference gene (16S) were designed using Primer-Uniprot and synthesized by IDT (S Table 4 in S1 File).

### qPCR conditions

qPCR was performed in technical triplicates (n = 3 biological replicates) on a BIO-RAD CFX96^TM^ Real-Time PCR System using PowerUp SYBR Green Master Mix (Thermo Fisher, Cat. No. A25741) at cycling conditions: 95°C/2 min; 40 cycles of 95°C/15 s, 60°C/60 s; followed by melt curve (65–95°C, 0.5°C increments).

### Data analysis

Relative expression was calculated by the 2^ − ΔΔCt method, with 16S as internal control and untreated samples as control/WT. Statistical analysis used Ordinary one-way ANOVA in GraphPad Prism v10.0 (p ≤ 0.05).

## Results

### Establishment of a stepwise colistin adaptation model

The MIC of WT/control *K. pneumoniae* clinical isolate was established for colistin using BMD method. *K. pneumoniae* (clinical isolates) resistant to varying MIC of colistin (1x MIC, 2x MIC and 4x MIC) were established *in-vitro* from the colistin sensitive (with MIC of WT/control was 0.25 µg/mL) *K. pneumoniae* clinical isolates using SDE of colistin to the pathogen(s). Briefly, *K. pneumoniae* cells (O.D.-based fixed inoculum, 10^6^ cells/mL) were exposed to 1x MIC for 4 hrs. (instead of 12–16 hrs.), reflecting a SDE and mimicking short-duration intake of antibiotics in real-life, and apparently, cell killing was observed in the culture. An aliquot of sample was plated on the MHA plate and incubated at 37°C overnight to retrieve survivors. These survivors were then isolated and propagated in broth culture without any antibiotic exposure for the next 3 days to de-stress the cells. The cycle was repeated unless the cells adapted to the selection pressure and the growth rate becomes comparable to the control/WT pathogen. Once the cells achieved comparable growth to the control, the cells were exposed to further increased antibiotic concentrations sequentially (2x followed by 4x MIC) to facilitate progressive adaptation under increasing antibiotic pressure. By following this workflow, the cells resistance to 1x, 2x, and 4x MIC were developed. The bacterial population exhibited hetero-resistance across successive passages, and growth was restored at higher antibiotic doses upon adaptation. A population with reduced susceptibility to colistin emerged because of this adaptation process, as evidenced by a steady rise in MIC [30,33–36].

### Validation of the established cell model of colistin-resistant *K. pneumoniae*

**Fig 1:**
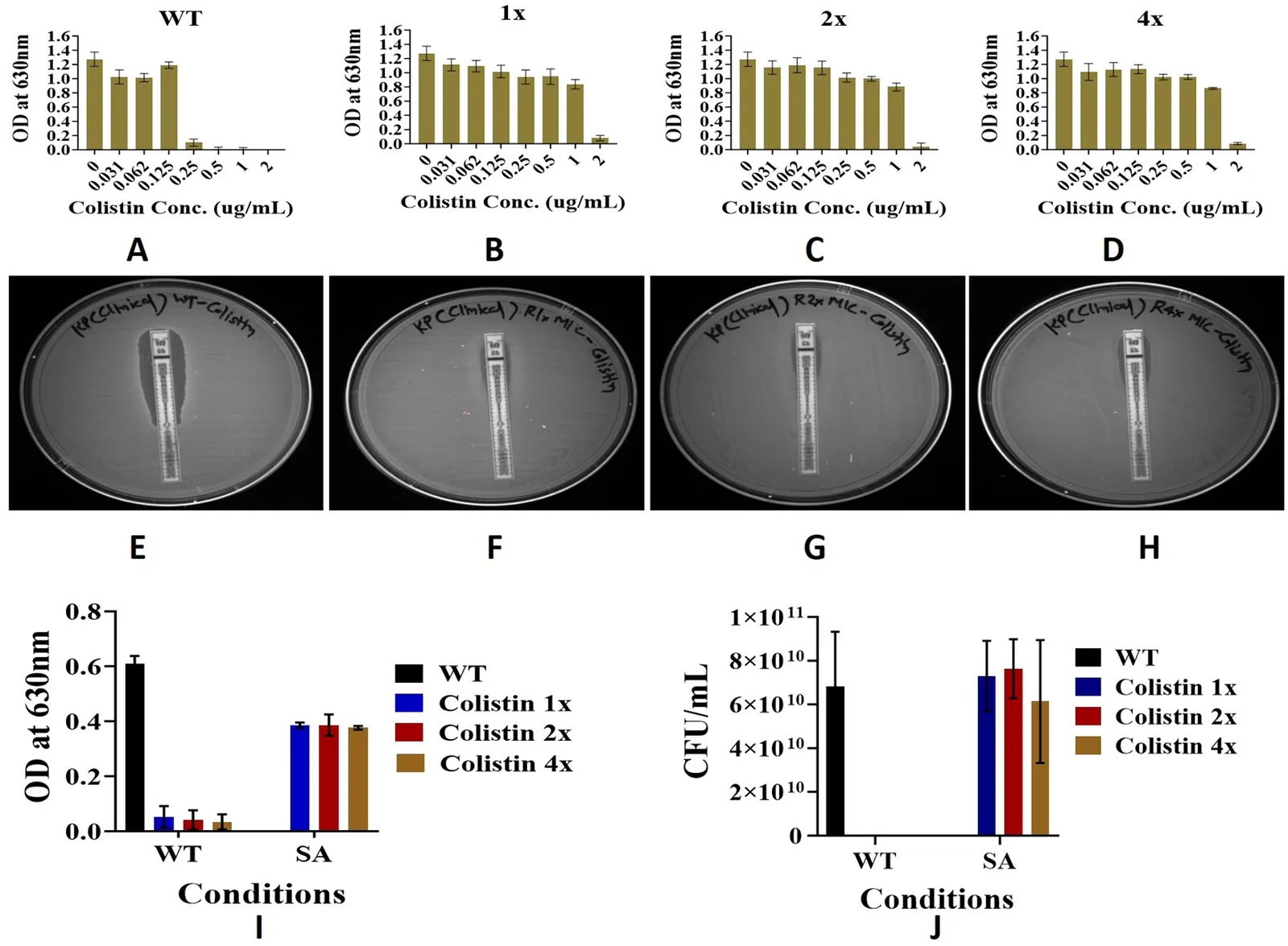
Validation of established model. MIC from broth microdilution methods from WT/control to adapted (A-D). E-strips based assay (E-H), and Absorbance and CFU-based validation 1x, 2x and 4x MIC adapted *K. pneumoniae* I and J respectively.

The established resistant *K. pneumoniae* cells were grown with or without respective antibiotic concentrations and evaluated for their MICs. The MIC of adapted pathogens was observed to be increased following stepwise antibiotic exposure, possibly due to the development of enduring resistance as an adaptive response (Fig 1A–1H). Control/WT strain showed no growth when exposed to 1x MIC, 2x and 4x doses indicating their antimicrobial sensitivity. However, the adapted cells (1x, 2x and 4x MIC adapted) were able to grow comparable to Control/WT strain when supplemented with respective antibiotic concentrations to which they were adapted. Interestingly, the MIC of cells adapted to 1xMIC increased to ~2 µg/mL (nearly 8-fold increase), which didn’t increase further even after exposure/adaptation to 2x MIC or 4x MIC. In adapted populations, repeated exposure to colistin led to the steady development of high-level colistin resistance (Fig 1I and 1J).

### Phenotypic characterization of adapted model: Pathogen identification and antibiotic susceptibility profiling

Once the stepwise antibiotic adaptation model was established, the integrity of the adapted bacterial population was confirmed by MALDI-TOF MS. All the adapted pathogens were identified as *K. pneumoniae* (confidence value >99.9%), confirming their original identity and no contamination or species-level variation during/after the adaptation process (S Table 1 in S1 File).

**Fig 2:**
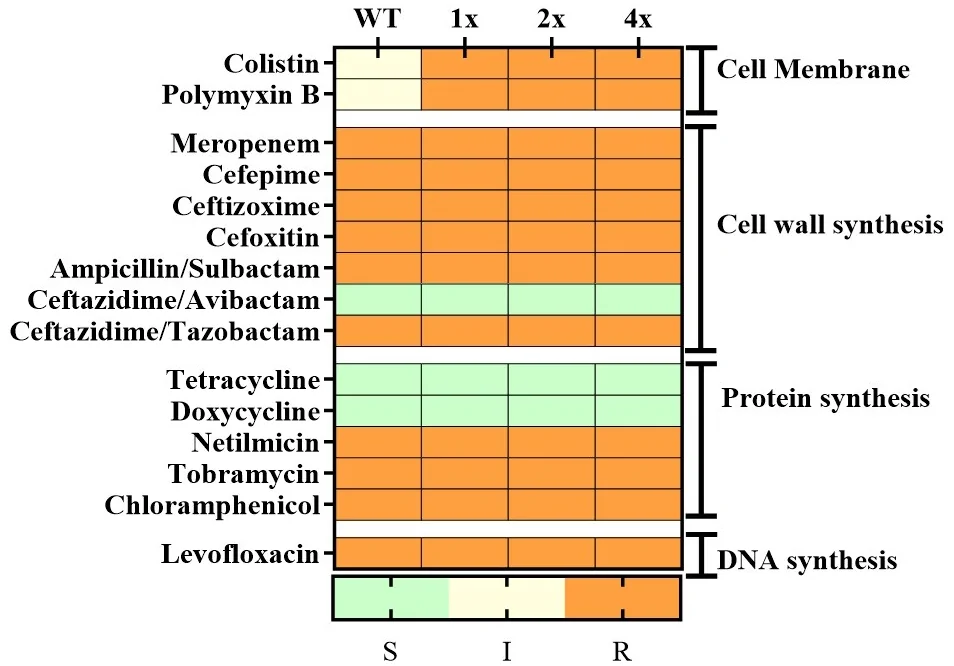
Antimicrobial susceptibility (AST) profile of resistant *K. pneumoniae* cells generated using an automated VITEK-2 system. Horizontal gaps among rows denote antibiotic categories based on mode of action. Vertical lines in the column denote different colistin resistant adapted to different antibiotics concentration (1x, 2x, and 4x MIC). S-Sensitive, I-Intermediate, and R-Resistance.

The VITEK-2 compact system with AST-card (AST N-407) was then utilized to determine their AST. The EUCAST criteria were used to automatically calculate and interpret MIC values. As observed in BMD, after stepwise exposure, the adapted population showed an increase in the MIC of the tested antibiotic (S Table 2 in S1 File). The adapted *K. pneumoniae* cells (1x, 2x, and 4x MIC) developed resistance against colistin and polymyxin B compared to its control/WT strain. Additionally, the adapted cells (1x, 2x, and 4x MIC) maintained their native (WT cells) resistance profile under all conditions. For example, they remained resistant to β-lactam antibiotics that mainly target cell wall synthesis (such as ampicillin/sulbactam, cefepime, ceftizoxime, cefoxitin, and meropenem), levofloxacin which inhibits nucleic acid synthesis, and antibiotics that target protein synthesis (such netilmicin, tobramycin, and chloramphenicol) (Fig 2).

### Global proteomic alterations underlying colistin resistance development in *Klebsiella pneumoniae*

**Fig 3:**
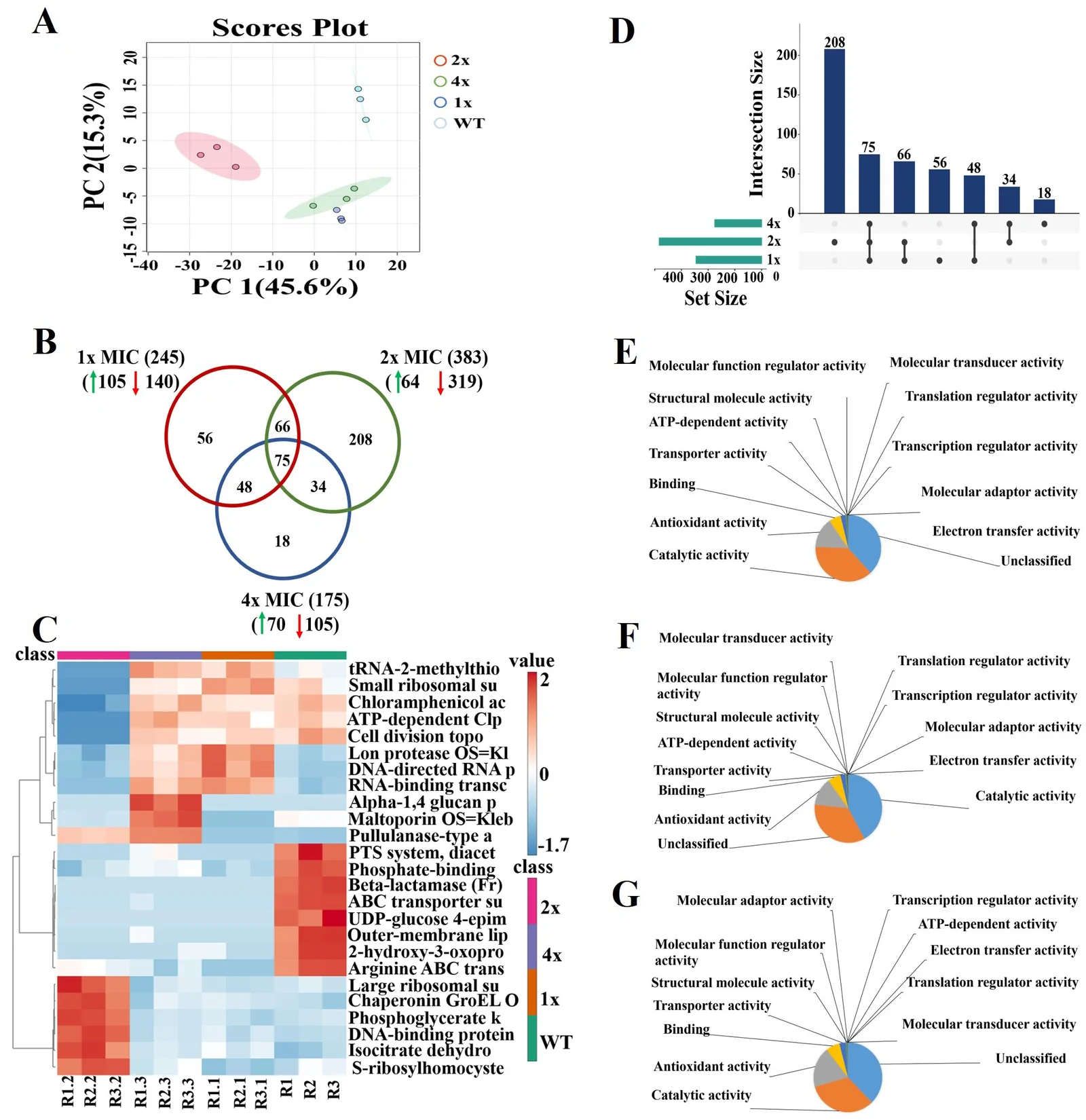
Multivariate and univariate analyses of global proteome changes. **(A)** Principal component analysis (PCA) score plots of the different stages depicting distinct clusters of adapted cells compared to control (WT) with 1x and 4x showing partial overlap. **(B)** Venn diagram showing the number of significantly affected proteins in different condition (1x, 2x, and 4x MIC compared to Control (WT)) along with shared-proteins among all adapted conditions. The up and down arrows indicate proteins that were significantly increased and decreased, respectively. (**C**) Hierarchical clustering of the top 25 differentially expressed shared proteins (FDR ≤ 0.05 and |log2 FC| ≥ 1, one-way ANOVA) demonstrated via heatmap profiles. Rows denote proteins, columns represent sample groups. The samples and proteins represented distinct clusters. (**D**) UpSet plots summarizing the distribution of significantly altered shared and unique proteins across all the adapted condition. Horizontal bars denote the total number of differentially expressed proteins in each group, whereas vertical bars indicate shared or unique protein subsets. DEPs were thereafter classified into molecular function categories using the PANTHER (Protein Analysis Through Evolutionary Relationships) database. High catalytic activity (GO:0003824) **(E)**, antioxidant activity (GO:0016209) **(F)** and binding (GO:0005488) **(G)** were observed to be enriched in all the adapted conditions.

Label-free quantitative proteomic profiles of colistin SDE-adapted *K. pneumoniae* populations (1x, 2x, and 4x MIC) were established to identify the proteomic alterations during the evolutionary trajectory of colistin-resistance. A total of 1379 proteins were identified in all conditions. To validate the proteomic results, a few genes (including upregulated [*arnA* (>2.5-fold) *and arnB* (>2.7-fold)]*,* and downregulated [*kphS* (3.3-fold)] from the top DEPs that were known to be associated with the emergence of AMR in the published literature, were examined at the transcriptional level using quantitative qRT-PCR. In line with the proteomic findings, the qRT-PCR results validated the expression patterns, i.e *arnA* and *arnB* were upregulated by ~4.5 and ~6 fold, respectively and *kphS* was 0.5-fold downregulated (S Fig 3 in S1 File). This agreement between gene expression and protein abundance enhances the reliability of the proteomic dataset.

PCA was then used to analyze the data in order to figure out the global proteome variation between the colistin SDE-adapted *K. pneumoniae* cells and the WT cells. The replicates of each experimental group clustered together, indicating the similar behaviour of the replicates during sample preparation and data acquisition strengthening the reliability of proteomic data. Distinct clusters of 2x MIC were established, while 1x and 4x showed partial overlap, indicating progressive proteome remodelling after stepwise colistin exposure (Fig 3A).

DEPs were determined by comparing the intensity of proteins in the adapted colistin-resistant cells to wildtype. A log2 fold change (log2FC) threshold of ≥ + 1 for upregulated proteins and ≤ −1 for downregulated proteins (corresponding to at least a ≥ 2-fold change) with a p-value of ≤0.05 to ensure statistical significance. At 1x MIC adapted resistant condition, a total of 245 DEPs (105 upregulated and 140 downregulated) were identified. In case of 2x MIC adapted resistant condition, a total of 383 DEPs (64 upregulated and 319 downregulated) DEPs were identified. Similarly, at 4x MIC adapted resistant condition, a total of 175 DEPs (70 upregulated and 105 downregulated) were identified (Fig 3B, Table 1, and S Fig 1 in S1 File). Venn diagram was used to illustrate the unique and common proteins among *K. pneumoniae* populations. Stage-specific expression trends in WT and adapted populations (1x, 2x, and 4x MIC) were observed across the increasing antibiotic concentrations. A total of 56 proteins were uniquely differentially expressed in 1x MIC adapted cells, whereas 208 were uniquely differentially expressed in 2x MIC adapted cells and 18 were uniquely differentially expressed in 4x MIC adapted cells (Fig 3B). The hierarchical clustering of the top 25 shared proteins also demonstrated distinct stage-wise clusters between WT and colistin SDE-adapted populations (1x, 2x, and 4x MIC), indicating proteome remodelling upon colistin exposure (Fig 3C). The overlapping DEPs among colistin SDE-adapted *K. pneumoniae* populations (1x, 2x, and 4x MIC) were identified using UpSet plot.

**Table 1:** DEPs proteins identified in *K. pneumoniae* for colistin at different antibiotic concentrations.

Conditions	Wild Type	1x MIC	2x MIC	4x MIC
Total Number of Identified Proteins	1379			
Differentially expressed Proteins (compared to control/WT)	NA	245	383	175
Upregulated		105	64	70
Downregulated		140	319	105
Unique proteins		56	208	18
Core Common proteins		75 (15 Up and 60 Down)		

A total of 75 DEPs (15 upregulated, 60 downregulated) were observed to be common among all the colistin SDE-adapted cells (1x, 2x, and 4x MIC). These common proteins most likely representing the core adaptive proteomic signature that is maintained throughout the sequential adaptation process at all MIC conditions and get impacted in the early phases of colistin exposure and hence may serve as early adaptive biomarkers. Contrarily, proteins found only at distinct MIC stages, probably representing stage-specific proteome alterations of each subsequent degree of colistin exposure (Fig 3B, D and S Table 3 in S1 File).

To understand the functional implications of altered proteomic changes associated with colistin SDE-adaptation, Gene Ontology (GO) functional enrichment of DEPs was carried out using the PANTHER (Protein Analysis Through Evolutionary Relationships) classification system. Molecular Function (MF) analysis demonstrated substantial enrichment of proteins linked to catalytic activity (GO:0003824), antioxidant activity (GO:0016209), and binding (GO:0005488) functions (Fig 3E-3G).

### Core common protein expression profiling, interaction network analysis highlights key functional modules underlying antibiotic adaptation

**Fig 4:**
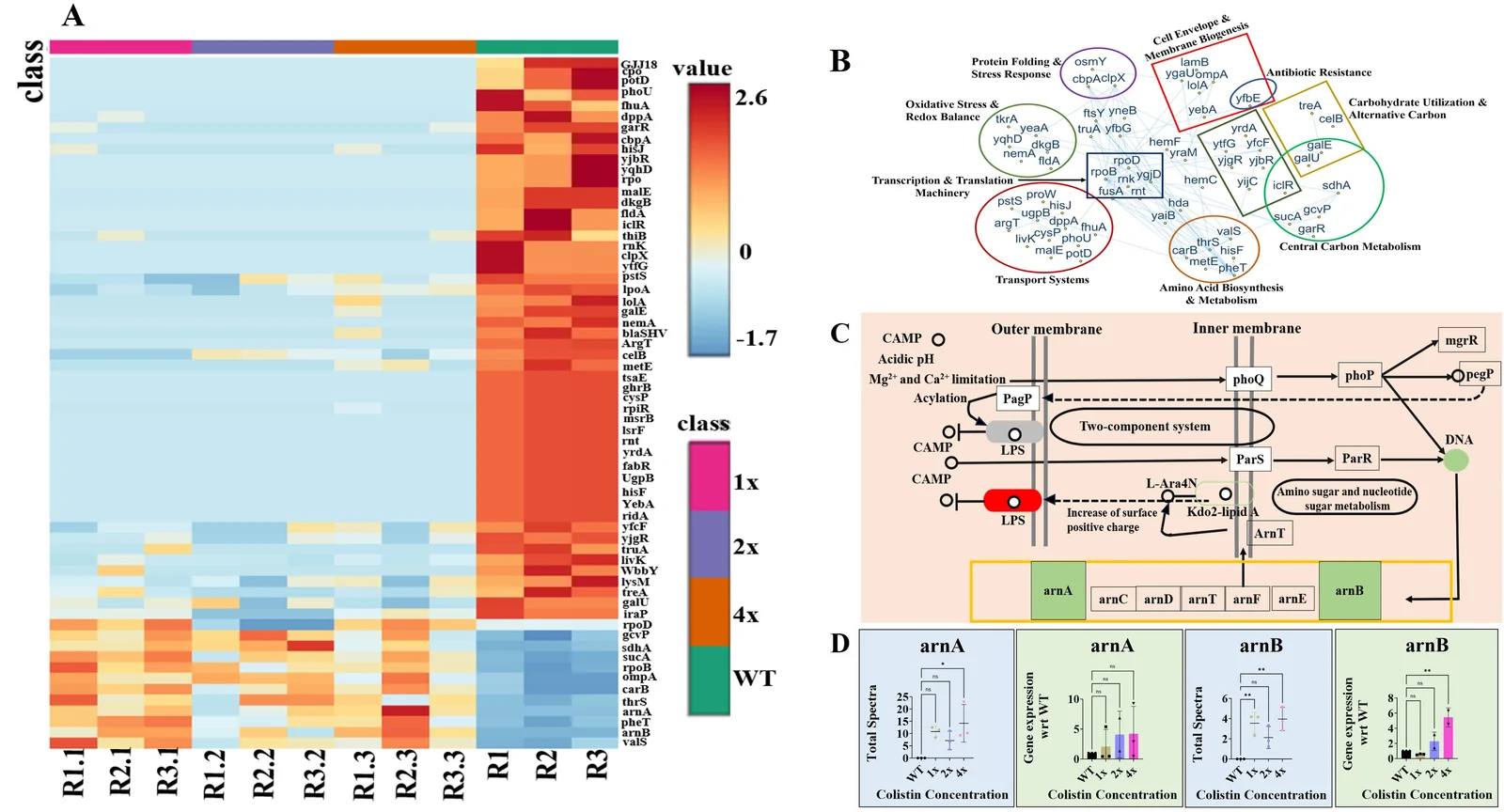
Analysis of identified 75 shared DEPs. **(A)** Heatmap of 75 shared DEPs across the adapted conditions. The heatmap demonstrates different expression dynamics throughout the experimental stages by displaying expression patterns and clustering of shared DEPs. The relative expression levels of proteins are displayed, with colour gradients denoting up-(Red) to down (Blue)-regulation at different stages. **(B)** The PPI network was constructed using the STRING database, which depicts the functional relationships among DEPs identified during colistin adaptation. Functional modules, such as cell membrane, membrane transport, stress response, and translation, were observed to be distinct and dominant clusters. **(C)** The roles of *arnA* and *arnB* in L-Ara4N synthesis are depicted in the KEGG-CAMP resistance pathway. *arnA* and *arnB* facilitates L-Ara4N incorporation into the lipid A which decreases its negative charge, reducing the ability of cationic antimicrobial peptides (colistin) to bind to it. **(D)** Upregulated protein expression (light blue) and gene expression (light green) of *arnA* and *arnB*. Validated upregulated *arnA* and *arnB* may be linked with the modulation of LPS biosynthesis or it’s modification contributing to reduced colistin susceptibility.

A total of 75 (15 upregulated and 60 downregulated) proteins, showed differentially expressed under all colistin SDE-adapted conditions (1x, 2x and 4x MIC), and hence were categorized based on published literature and their reported biological functions that may be involved in the emergence of AMR. A number of proteins involved in such activated were differentially expressed among all the tested conditions; lipid A modification and colistin resistance machinery (*arnA,arnB, phoU,* etc.), membrane transporters and envelope adaptation (*potD, gsiB, pstS* etc.), oxidative stress defense and detoxification (*gst, nemA, ghrB* etc.), global transcriptional regulations and stress response regulated *(rsd, rpoD, rpoB,* etc.), energy metabolism and metabolic rewiring (*sdhA, sucA, gcvP,* etc.), protein synthesis, RNA processing, and translational adaptation (*fusA, thrS, pheT, valS,* etc.), quorum sensing, persistence, and adaptive survival (*lsrF, ftsY,*etc.), classical antibiotic resistance-associated proteins (*blaCTX-M*), and miscellaneous (*fhuA_2, garR_1, ftsY,* etc.). Thereafter, a heatmap was generated to investigate the expression dynamics of conserved proteins between the WT and adapted populations. Distinct clustering patterns were observed, indicating stage-specific regulation of essential cellular processes and adaptive pathways following antibiotic exposure (Fig 4A). Furthermore, a PPI network was constructed using the STRING database to gain a more comprehensive understanding of the biological processes/pathways underlying antimicrobial adaptation. Various functional clusters, that may be involved in emergence of AMR, were identified that included proteins related to transport systems (*potD, fhuA, phoU,* etc.), cell envelope and membrane biogenesis (*arnA, arnB,* etc.), oxidative stress and redox balance response (*msrB, nemA,* etc.), central carbon metabolism (TCA, Glyoxylate, Energy), amino acid biosynthesis and metabolism (*sdhA, sucA,* etc.), and transcription and translation machinery (*rpoD, rpoB,* etc.) (Fig 4B).

### Integrated proteomic analysis, KEGG based pathway mapping, and qRT-PCR validation of *arnA/arnB* genes

Among the DEPs, *valS, arnA, arnB, pheT,* and *thrS* were top most upregulated DEPs while *gjJ18, kphS, B5L96, potD*, and *phoU* were downregulated DEPs. *arnA* (bifunctional polymyxin resistance protein) and *arnB* (UDP-4-amino-4-deoxy-L-arabinose--oxoglutarate aminotransferase), are known to modify lipid A. Together, they enable the addition of positively charged L-Ara4N to lipid A in the LPS, reducing the negative charge and hence colistin binding to *K. pneumoniae* cells. Conversely, *potD, phoU,* and *valS* are associated with stress response. Glutathione S-transferase (*GJJ18*) detoxifies reactive oxygen species (ROS) and epoxides generated by colistin-induced membrane damage, while non-heme chloroperoxidase (*KPHS*) may mitigate oxidative stress through halogenation or peroxidase activity. ABC transporters like *PotD* (polyamine-specific) and the hydroxymethylpyrimidine substrate-binding protein (*B5L96*, linked to vitamin B1/thiamine uptake) support metabolic shifts for persistence under stress, and *PhoU* fine-tunes phosphate transport to activate PhoPQ TCS for LPS modification (S Fig 2 in S1 File).

After validating the expression of two highly elevated proteins (*arnA* and *arnB*) with known functions in AMR at the transcriptional level, they were mapped onto KEGG pathways to obtain a mechanistic understanding of their functional involvement. Their coordinated upregulation was associated with modulation of various key biological pathways (i.e., cellular adaptation, metabolic regulation), that plays role in emergence of AMR (Fig 4C, 4D).

## Discussion

Antimicrobial Resistance (AMR) has become one of the major escalating threats to global public health [1]. Colistin is a last-resort antibiotic used for treating MDR/XDR *K. pneumoniae* infections. However, as the use increasing, the prevalence of colistin-resistant pathogens is also increasing and posing a significant risk of it becoming ineffective, necessitating development of new strategies to treat antibiotic-resistant infections and preserve antibiotics for future use [37,38]. Besides the right dose and antibiotic, the short-duration exposure (SDE) of antibiotics is among the most common situations in the real world that contribute to the emergence of AMR. Hence, improved understanding the mechanisms of SDE-induced emergence of antibiotic-resistance can help in devising novel and effective approaches to treat/manage such drug-resistant pathogens.

Therefore, in this investigation, we have tried to mimic the real-world like situation (SDE to antibiotics) and developed SDE-induced colistin (1x, 2x and 4x MIC)-resistant *K. pneumoniae* cells using repeated cycles of short-duration (4 hrs., instead ≥16 hrs.) exposure of colistin followed by enrichment of survivors till they develop resistance.

Interestingly, the MIC of 1x MIC-adapted cells increased to ~2 µg/mL (nearly 8-fold increase) through transient adaptive response and remained unchanged even after SDE exposure and adaptation to 2x MIC or 4x MIC. This suggests a non-linear adaptive response rather than a progressive stepwise pattern for the emergence of antibiotic resistance. SDE to 1x MIC colistin was sufficient to trigger rapid physiological remodelling, potentially via altering membrane homeostasis, molecular targets and cellular metabolism and evolve to achieve antibiotic resistance [39,40]. This is in-line with the notion that adaptive tolerance can precede and potentially facilitate the emergence (polymyxin-mediated) of AMR [16,36,41,42].

The SDE-induced colistin-resistant *K. pneumoniae* cells were proteome profiled using LC-MS and a total of 1379 proteins were identified. PCA shows distinct cluster of WT and 2x MIC, whereas partial overlap between the 1x and 4x MIC-adapted populations indicating that proteome remodelling is dynamic rather than linear during sequential colistin adaption. A transitional adaptive state characterized by extensive physiological reprogramming in response to increasing antibiotic pressure may be represented by distinctive clustering of the 2x MIC population. The partial overlap between the 1x and 4x populations, on the other hand, may be indicating the survival of a core adaptive proteomic signature that is formed during early adaptation and sustained after the resistant phenotype is stabilized [43,44]. The number of DEPs was initially increased (383 in the 2x MIC colistin-resistant *K. pneumoniae*) and later decreased (175 in the 4x MIC colistin-resistant *K. pneumoniae*) compared to 1x MIC colistin-resistant *K. pneumoniae* (245 DEPs). A similar number of DEPs (315, consisting of 133 upregulated and 182 downregulated) between the polymyxin B-resistant *vs*. sensitive *K. pneumoniae* have been reported in previous study, supporting the number of identified DEPs (245) in our study [24]. The 2x MIC colistin-resistant *K. pneumoniae* represented increased numbers of DEPs and unique DEPs compared to 1x MIC resistant population, possibly by triggering extensive physiological and metabolic reprogramming to maintain cellular homeostasis under increased antibiotic molecular stress. However, why the number of DEPs and unique DEPs in 4x MIC resistant cells came down again, despite the MIC of the cells was unchanged, may indicate the potential subsiding of the dose/stage-specific DEPs, though the reason remains unclear. Of these, 75 DEPs (15 upregulated and 60 downregulated) were common among all antibiotic concentrations (1x, 2x and 4x MIC), indicating potentially a conserved adaptive response against colistin, instead dose/stage (MIC)-specific alterations. All of these proteins showed unidirectional (down-regulated or upregulated) significantly differential expression at the stringent criteria (log2FC, *p-value*: ≤ 0.05) in all the adapted conditions (1x, 2x and 4x MIC) which may potentially be an indicator of no/low carry-over effect in DEPs from one dose to another, instead they are the dose-dependent response from the cells/pathogens; however functional investigation may help to eliminate/minimize the carry over effect. Subsequently, their biological relevance (75 DEPs) was assessed in the modulation of lipid A synthesis/maintenance, membrane transporters and envelope adaptation and quorum sensing that potentially constitute the adaptive survival strategies and help the pathogen to develop resistance against colistin [9,27,43]. However, unlike the previous study, which compared only susceptible and fully resistant populations, our sequential stepwise adaptive evolution model identified persistent adaptive protein alterations that remains maintained throughout resistance evolution, as well as emerges in stage/dose-specific manner, thereby providing temporal insight into the progressive acquisition of colistin resistance [24].

DEPs were further analysed for gene ontology (GO) and observed to be significantly enriched in molecular functions associated with catalytic activity (GO:0003824), antioxidant activity (GO:0016209) and binding activity (GO:0005488). Similarly, PPI network also showed DEPs clusters associated with cell envelope and membrane biogenesis, oxidative stress and redox balance response, central carbon metabolism (TCA, Glyoxylate, Energy), amino acid biosynthesis and metabolism, and transcription and translation machinery, indicating a coordinated regulation of multiple cellular processes rather than isolated alterations to molecules. The metabolic reprogramming, such as target modification, energy dynamics, central carbon metabolism and oxidative stress, is already known to lead to decreased antibiotic sensitivity [23,41,43,45,46].

Furthermore, *valS, arnA, arnB, pheT,* and *thrS* were identified as the top 5 upregulated DEPs, whereas, *gjJ18, kphS, B5L96, potD*, and *phoU* were the top 5 downregulated DEPs. *arnA* and *arnB* are the crucial enzymes in the *arnBCADTEF* (or *pmrHFIJKLM*) operon, which adds 4-amino-4-deoxy-L-arabinose (L-Ara4N) to lipid A and reduces the negative charge of the LPS. By reducing the negative charge of lipid A, this cationic alteration stops colistin from binding to the LPS and leads to the emergence of AMR [47,48]. Besides, *phoU* mutations or dysregulation leads to constitutive activation of phoPQ, upregulating the arn operon for LPS modification and reducing colistin binding [49–52]. This investigation reports a direct correlation between the levels of colistin resistance and the higher Arn protein content in colistin-resistant bacteria and is in-line with the existing research [52–54]. A periplasmic binding protein for the spermidine/putrescine ABC transporter *(PotABCD),* which is involved in polyamine uptake and promotes bacterial growth and stress adaptability, is encoded by *potD.* Some colistin-resistant proteomic profiles have shown upregulation of *potD,* helping in maintaining the membrane integrity or mitigate the effects of cationic peptides [55–57]. However, we observed down-regulation of *potD* in colistin-resistant bacteria which may be further promoting the decreased colistin binding to the membrane and the emergence of AMR; hence is consistent with published research [52,58]. Similarly, although there is no information (particularly on colistin), kphS homologs may contribute to both indirect resistance and pathogenicity by oxidative stress, efflux, or membrane remodeling [59]. ValS encodes valyl-tRNA synthetase, which is required for protein synthesis by charging tRNA with valine. Both phenylalanyl-tRNA synthetase (*PheT*) and threonyl-tRNA synthetase (*ThrS*) are essential for aminoacylation in translation. However, according to reviewed genomic and proteomic studies, there is no literature evidence that connect their expression alterations in *K. pneumoniae* to colistin resistance. Similarly, *gjJ18* and *B5L96* appear as hypothetical or strain-specific locus tags with no direct evidence connecting expression alterations in *K. pneumoniae* to colistin resistance.

Further, the reliability of the observed molecular and phenotypic alterations is strengthened by a correlation between proteomic data and qRT-PCR validation of the *arnA, arnB*, and *kphS* genes in our study as well as matching with the published literature [43]. ⁠KEGG Cationic antimicrobial peptide (CAMP) resistance pathway mapping provided further insight into the molecular mechanisms underlying colistin adaptation in *K. pneumoniae*. The DEPs (arnA, arnB), which were mostly associated with pathways involved in LPS alterations and stress response, demonstrated a coordinated cellular response to antibiotic pressure and in-line with previous observations with an antimicrobial peptide (PaDBS1R1) [24,25]. Collectively, these pathway-level changes show that colistin adaptability is driven by integrated regulation of membrane structure, transport systems, and metabolic networks. Such coordinated reactions may facilitate the shift from transient tolerance to stable resistance over extended antibiotic exposure and most likely result in reduced colistin sensitivity. However, to determine whether these proteins directly contribute to colistin resistance, tolerance, or persistence, further functional and mechanistic research is needed to establish their role in the emergence of colistin-resistance, tolerance, or persistence.

Limitations of the study: There are some limitations associated with this study, which open a new area of investigation –

1) Only a single clinical isolate of *K. pneumoniae* along with colistin was used in this study. Inclusion of more Gram-negative/positive pathogens and antibiotics can further strengthen the findings of this study.
2) Inclusion of transcriptomics and metabolomics data for the adapted bacterial population representing resistance and the emergence of AMR may further explain the differential regulatory layers among proteins and/or gene level driven by protein expression changes.
3) Pathways and biomolecular associations were concluded based on curated databases and literature and hence functional validation of the observations was not performed in the investigator’s laboratory.

Overall, our results provide a comprehensive proteomic understanding of cell membrane reprogramming following colistin treatment in *K. pneumoniae* or during different stages of AMR emergence. The study offers new perspectives on adapted proteome alteration/mechanisms under the SDE condition for emergence of AMR.

## Supporting information

S1 File Supporting figures and tables. This file contains**-** S Table 1: Pathogen Identification by MALDI-TOF MS, S Table 2: BioMerieux VITEK 2 AST Card No. N407 Antibiotics Details, S Table 3: Core common DEPs proteins among all the adapted conditions, S Table 4: Gene and their primer sequence detail for Quantitative RT-PCR, S Figure 1: Volcano plots showing DEPs in different adapted *K. pneumoniae* In 1x MIC- 245 DEPs (105 upregulated and 140 downregulated), In 2x MIC – 383 (64 upregulated and 319 downregulated), and In 4x MIC – 175 (70 upregulated and 105 downregulated), S Figure 2: Core common DEPs expression profiling of 75 (15 upregulated and 60 downregulated) proteins, S Figure 3: Quantitative RT PCR gene expression profiling of *arnA*, *arnB* (upregulated) and *kphS* (downregulated).
(DOCX)

S2 File Supporting excel contain the information of total identified proteins total spectra, Accession Number, Alternate ID (gene name), Molecular Weight, Protein Grouping Ambiguity, Taxonomy. (XLS)

## Abbreviations

AMR: Antimicrobial resistance
MIC: Minimum Inhibitory Concentration
WHO: World Health Organization
DBT: Department of Biotechnology
ESKAPE: Enterococcus faecium, Staphylococcus aureus, Klebsiella pneumoniae, Acinetobacter baumannii, Pseudomonas aeruginosa, and Enterobacter spp.
MDR/XDR: Multi Drug-Resistance/ Extensively Drug-Resistant
WT: Wild Type
LPS: Lipopolysaccharides
CAMP: Cationic antimicrobial peptide
MHA/B: Muller Hinton Agar/Broth
BMD: Broth Micro Dilution
CLSI: Clinical Laboratory Standards Institute
AST: Antimicrobial Susceptibility Test
MALDI-TOF: Matrix Assisted Laser Desorption Ionization-Time of Flight
LC-MS/MS: Liquid Chromatography–Tandem Mass Spectrometry
DEPs: Differential Expression Proteins
KP: *Klebsiella pneumoniae*
PBS: Phosphate Buffered Saline
KEGG: Kyoto Encyclopaedia of Genes and Genomes
OD: Optical Density
