# Supplementary material for: Proteome-wide remodelling in Klebsiella pneumoniae during step-wise adaptive evolution against colistin
Source: PLoS One. 2026 Sep 24;21(9):e0358791. doi: 10.1371/journal.pone.0358791 (PMC13607800; doi:10.1371/journal.pone.0358791)
Supplement: Supplementary file 1 [file pone.0358791.s001.docx]

**S Table 1**. Pathogen Identification by MALDI-TOF MS.

| **Patient ID** | **Patient Name** | **ID** | **Specimen Type** | **Organism Name** | **Confidence Value** |
| --- | --- | --- | --- | --- | --- |
| 1 | Clinical WT | T-11-1 | Bacterial Colony | *K. pneumoniae* | 99.99% |
| 2 | Clinical 1x MIC Adapted | T-12-1 | Bacterial Colony | *K. pneumoniae* | 99.99% |
| 3 | Clinical 2x MIC Adapted | T-13-1 | Bacterial Colony | *K. pneumoniae* | 99.99% |
| 4 | Clinical 4x MIC Adapted | T-14-1 | Bacterial Colony | *K. pneumoniae* | 99.99% |

**S Table 2**.- BioMerieux VITEK 2 AST Card No. N407 Antibiotics Details

|  | **BioMerieux VITEK 2 AST Card No. N407 Antibiotics Details** | | | |
| --- | --- | --- | --- | --- |
| **S.No.** | **Antibiotics** | **Code** | **Mode of action** | **Class** |
| **1** | **Ampicillin/Sulbactam** | SAM | Cell wall synthesis | Aminopenicillins |
| **2** | **Cefoxitin** | FOX | Cell wall synthesis (PBP) | Cephamycin |
| **3** | **Ceftizoxime** | CZX | Cell wall synthesis (PBP) | Third-Generation Cephalosporin |
| **4** | **Ceftazidime/Avibactam** | CZA | Cell wall synthesis (PBP) | Third-Generation Cephalosporin |
| **5** | **Ceftazidime/Tazobactam** | CT | Cell wall synthesis (PBP3) | Third-Generation Cephalosporin |
| **6** | **Cefepime** | FEP | Cell wall synthesis | Fourth-Generation Cephalosporin |
| **7** | **Meropenem** | MEM | Cell wall synthesis (PBP) | Carbapenems |
| **8** | **Netilmicin** | NET | Protein synthesis (30S) | Aminoglycoside Antibiotic |
| **9** | **Tobramycin** | TM | Protein synthesis (30S-50S) | Aminoglycoside Antibiotic |
| **10** | **Levofloxacin** | LEV | Bacterial DNA synthesis GyrB | Fluoroquinolones |
| **11** | **Doxycycline** | DO | Protein synthesis (30S) | Tetracycline antibiotics |
| **12** | **Tetracycline** | TE | Protein synthesis (30S) | Tetracycline antibiotics |
| **13** | **Chloramphenicol** | C | Protein synthesis (50S) | Amphenicol Antibiotic |
| **14** | **Colistin** | CS | Cell Membrane | Polymyxin Antibiotic |
| **15** | **Polymyxin B** | PB | Cell Membrane | Polymyxin Antibiotic |

**S Table 3**. Core common DEPs proteins among all the adapted conditions.

| **Common Identified Proteins (75)** | **Gene name** | **1x MIC (log2FC)** | **2x MIC**  **(log2FC)** | **4x MIC**  **(log2FC)** |
| --- | --- | --- | --- | --- |
| **Glutathione S-transferase OS=Klebsiella pneumoniaeOX=573** | ***GJJ18*** | -3.6101 | -3.6101 | -3.6101 |
| **Non-heme chloroperoxidase OS=Klebsiella pneumoniaesubsp. pneumoniae (strain HS11286) OX=1125630** | ***KPHS_35090*** | -3.386 | -3.386 | -3.386 |
| **Hydroxymethylpyrimidine ABC transporter substrate-binding protein OS=Klebsiella pneumoniaeOX=573** | ***B5L96_27030*** | -3.386 | -3.386 | -3.386 |
| **ABC transporter OS=Klebsiella pneumoniaeOX=573** | ***potD*** | -3.386 | -3.386 | -3.386 |
| **Phosphate-specific transport system accessory protein PhoU OS=Klebsiella pneumoniae30660/NJST258_1 OX=1420012** | ***KPNJ1_05478*** | -3.285 | -3.285 | -3.285 |
| **Ferrioxamine receptor OS=Klebsiella pneumoniaeOX=573** | ***fhuA_2*** | -3.2835 | -3.2835 | -3.2835 |
| **Porphobilinogen deaminase OS=Klebsiella pneumoniaeOX=573** | ***hemC*** | -3.2835 | -3.2835 | -3.2835 |
| **Dipeptide-binding ABC transporter, periplasmic substrate-binding component OS=Klebsiella pneumoniaeOX=573** | ***gsiB*** | -3.2764 | -2.7137 | -2.7137 |
| **2-hydroxy-3-oxopropionate reductase OS=Klebsiella pneumoniaeOX=573** | ***garR_1*** | -3.1435 | -2.5569 | -3.1927 |
| **Osmotically inducible protein Y OS=Klebsiella pneumoniae30660/NJST258_1 OX=1420012** | ***KPNJ1_04773*** | -2.9667 | -2.9667 | -3.3987 |
| **Curved DNA-binding protein OS=Klebsiella pneumoniaeOX=573** | ***cbpA*** | -2.8826 | -2.8826 | -2.8826 |
| **Histidine-binding protein OS=Klebsiella pneumoniae30660/NJST258_1 OX=1420012** | ***KPNJ1_01641*** | -2.8533 | -2.9278 | -3.0206 |
| **Protein yjbR OS=Klebsiella pneumoniaeOX=573** | ***yjbR_2*** | -2.7949 | -2.7949 | -2.7949 |
| **Aldo/keto reductase OS=Klebsiella pneumoniaeOX=573** | ***iolS_5*** | -2.7949 | -2.7949 | -2.7949 |
| **Regulator of sigma D OS=Klebsiella pneumoniaeOX=573** | ***rsd*** | -2.7948 | -2.7948 | -2.7948 |
| **Maltodextrin-binding protein OS=Klebsiella pneumoniaeOX=573** | ***cycB*** | -2.7947 | -2.7947 | -2.7947 |
| **2,5-diketo-D-gluconic acid reductase OS=Klebsiella pneumoniae30660/NJST258_1 OX=1420012** | ***KPNJ1_00736*** | -2.7947 | -2.7947 | -2.7947 |
| **Flavodoxin OS=Klebsiella pneumoniaesubsp. pneumoniae (strain HS11286) OX=1125630** | ***KPHS_42100*** | -2.7945 | -2.7945 | -2.7945 |
| **Acetate operon repressor OS=Klebsiella pneumoniaeIS39 OX=1432561** | ***IclR-family acetate operon*** | -2.7945 | -2.7945 | -2.7945 |
| **Thiamine-binding periplasmic protein OS=Klebsiella pneumoniaesubsp. pneumoniae OX=72407** | ***E9161_11450*** | -2.7519 | -3.5112 | -2.9022 |
| **NAD dependent epimerase/dehydratase family OS=Klebsiella pneumoniae(strain 342) OX=507522** | ***KPK_0762*** | -2.6999 | -2.6999 | -2.6999 |
| **Nucleoside diphosphate kinase regulator OS=Klebsiella pneumoniaeOX=573** | ***CK244_002910*** | -2.6999 | -2.6999 | -2.6999 |
| **ATP-binding protein OS=Klebsiella pneumoniaesubsp. pneumoniae OX=72407** | ***GLO21_027010*** | -2.6999 | -2.6999 | -2.6999 |
| **Oxidoreductase OS=Klebsiella pneumoniaeOX=573** | ***ETE75_19105*** | -2.6711 | -1.5503 | -1.3143 |
| **Glycine betaine/choline ABC transporter membrane protein OS=Klebsiella pneumoniaesubsp. pneumoniae (strain HS11286) OX=1125630** | ***KPHS_36310*** | -2.6603 | -2.9117 | -2.2217 |
| **Phosphate-binding protein PstS OS=Klebsiella pneumoniaeOX=573** | ***pstS*** | -2.6018 | -2.4267 | -1.239 |
| **Penicillin-binding protein activator LpoA OS=Klebsiella pneumoniaesubsp. pneumoniae OX=72407** | ***lpoA*** | -2.5995 | -2.5995 | -2.0314 |
| **Outer-membrane lipoprotein carrier protein OS=Klebsiella pneumoniae30660/NJST258_1 OX=1420012** | ***lolA*** | -2.5993 | -2.5993 | -3.0314 |
| **UDP-glucose 4-epimerase OS=Klebsiella pneumoniaeOX=573** | ***galE*** | -2.5698 | -2.5698 | -2.5698 |
| **N-ethylmaleimide reductase OS=Klebsiella pneumoniaeOX=573** | ***nemA*** | -2.5278 | -2.5278 | -2.5448 |
| **Maltoporin OS=Klebsiella pneumoniaeOX=573** | ***bglH_7*** | -2.5127 | -2.5127 | -2.0205 |
| **Beta-lactamase (Fragment) OS=Klebsiella pneumoniaeOX=573** | ***blaCTX-M*** | -2.5127 | -2.5127 | -2.5127 |
| **Arginine ABC transporter, periplasmic arginine-binding protein ArtI OS=Klebsiella pneumoniaesubsp. pneumoniae (strain HS11286) OX=1125630** | ***KPHS_17730*** | -2.4908 | -1.3668 | -2.2158 |
| **PTS system, diacetylchitobiose-specific IIB component OS=Klebsiella pneumoniae30660/NJST258_1 OX=1420012** | ***KPNJ1_03279*** | -2.4908 | -2.4908 | -2.0491 |
| **Cytosolic protein OS=Klebsiella pneumoniae30660/NJST258_1 OX=1420012** | ***KPNJ1_03500*** | -2.4469 | -2.7325 | -1.7661 |
| **5-methyltetrahydropteroyltriglutamate--homocysteine methyltransferase OS=Klebsiella pneumoniae30660/NJST258_1 OX=1420012** | ***KPNJ1_02305*** | -2.3734 | -2.3734 | -2.3734 |
| **tRNA threonylcarbamoyladenosine biosynthesis protein TsaE OS=Klebsiella pneumoniaeOX=573** | ***tsaE*** | -2.3734 | -2.3734 | -2.3734 |
| **Glyoxylate/hydroxypyruvate reductase B OS=Klebsiella pneumoniae30660/NJST258_1 OX=1420012** | ***ghrB*** | -2.3734 | -2.3734 | -2.3734 |
| **ABC transporter substrate-binding protein OS=Klebsiella pneumoniaeOX=573 GN=AI2671V1_2079** | ***AI2671V1_2079*** | -2.3734 | -2.3734 | -4.3903 |
| **Transcriptional regulator, RpiR family protein OS=Klebsiella pneumoniae30660/NJST258_1 OX=1420012** | ***KPNJ1_04934*** | -2.3733 | -2.3733 | -2.3733 |
| **Inner membrane protein OS=Klebsiella pneumoniaeOX=573** | ***SAMEA4873563_00613*** | -2.3733 | -2.3733 | -2.3733 |
| **Probable haloacid dehalogenase-like hydrolase STY3852 OS=Klebsiella pneumoniaeIS53 OX=1432554** | ***N/A*** | -2.3733 | -2.3733 | -2.3733 |
| **Free methionine-R-sulfoxide reductase OS=Klebsiella pneumoniaeOX=573** | ***msrC*** | -2.3733 | -2.3733 | -2.3733 |
| **Autoinducer 2 (AI-2) aldolase LsrF OS=Klebsiella pneumoniaeIS43 OX=1432552** | ***LsrF*** | -2.3733 | -2.3733 | -2.3733 |
| **Ribonuclease T OS=Klebsiella pneumoniaeOX=573** | ***rnt*** | -2.3733 | -2.3733 | -2.3733 |
| **Carbonic anhydrase, family 3 OS=Klebsiella pneumoniaeOX=573** | ***yrdA_2*** | -2.3733 | -2.3733 | -2.3733 |
| **HTH-type transcriptional repressor FabR OS=Klebsiella pneumoniae30660/NJST258_1 OX=1420012** | ***fabR*** | -2.3733 | -2.3733 | -2.3733 |
| **Glycerol-3-phosphate ABC transporter OS=Klebsiella pneumoniaeOX=573** | ***lacE_1*** | -2.3733 | -2.3733 | -2.3733 |
| **Imidazole glycerol phosphate synthase subunit HisF OS=Klebsiella pneumoniaeOX=573** | ***hisF*** | -2.3733 | -2.3733 | -2.3733 |
| **Peptidoglycan-specific endopeptidase, M23 family protein OS=Klebsiella pneumoniae30660/NJST258_1 OX=1420012** | ***KPNJ1_01138*** | -2.3733 | -2.3733 | -2.3733 |
| **RidA family protein OS=Klebsiella pneumoniaeOX=573** | ***CK244_023850*** | -2.3733 | -2.3733 | -2.3733 |
| **Glutathione S-transferase OS=Klebsiella pneumoniaeOX=573** | ***gst*** | -2.3537 | -2.0863 | -1.5149 |
| **DUF853 domain-containing protein OS=Klebsiella pneumoniaeOX=573** | ***D1220_21855*** | -2.0925 | -2.6666 | -2.0952 |
| **Pseudouridine synthase OS=Klebsiella pneumoniae30660/NJST258_1 OX=1420012** | ***KPNJ1_01364*** | -2.046 | -2.7081 | -2.7081 |
| **Amino acid ABC transporter substrate-binding protein OS=Klebsiella pneumoniaesubsp. pneumoniae OX=72407** | ***E9161_07935*** | -1.95 | -2.4504 | -2.023 |
| **Uncharacterized protein OS=Klebsiella pneumoniaeOX=573** | ***KPZU09_42010*** | -1.8601 | -2.5992 | -1.3243 |
| **Sugar-binding protein OS=Klebsiella pneumoniae30660/NJST258_1 OX=1420012** | ***KPNJ1_04102*** | -1.7629 | -2.5993 | -2.5993 |
| **Peptidoglycan binding protein (LysM domain) OS=Klebsiella pneumoniae30660/NJST258_1 OX=1420012** | ***KPNJ1_02757*** | -1.6318 | -1.8135 | -1.3245 |
| **Periplasmic trehalase OS=Klebsiella pneumoniaeOX=573** | ***treA_1*** | -1.5864 | -2.8954 | -2.4645 |
| **UDP-sugar diphosphatase OS=Klebsiella pneumoniae30660/NJST258_1 OX=1420012** | ***KPNJ1_04226*** | -1.2758 | -1.2615 | -2.5949 |
| **Signal recognition particle receptor FtsY OS=Klebsiella pneumoniae30660/NJST258_1 OX=1420012** | ***ftsY*** | -1.2636 | -2.656 | -1.1378 |
| **Anti-adapter protein IraP OS=Klebsiella pneumoniae30660/NJST258_1 OX=1420012** | ***iraP*** | -1.2347 | -2.4444 | -1.2359 |
| **RNA polymerase sigma factor RpoD OS=Klebsiella pneumoniaesubsp. pneumoniae (strain HS11286) OX=1125630** | ***rpoD*** | 1.1986 | -1.8229 | 1.0057 |
| **Elongation factor G OS=Klebsiella pneumoniae30660/NJST258_1 OX=1420012** | ***fusA*** | 1.2584 | 1.4761 | 1.1326 |
| **Glycine dehydrogenase (decarboxylating) OS=Klebsiella pneumoniaeOX=573** | ***gcvP*** | 1.5724 | 1.5282 | 1.2959 |
| **Succinate dehydrogenase flavoprotein subunit OS=Klebsiella pneumoniaeOX=573** | ***sdhA*** | 1.5868 | 1.7663 | 1.2325 |
| **oxoglutarate dehydrogenase (succinyl-transferring) OS=Klebsiella pneumoniaeOX=573** | ***sucA*** | 1.7813 | 1.2194 | 1.3918 |
| **DNA-directed RNA polymerase subunit beta OS=Klebsiella pneumoniaeOX=573** | ***rpoB*** | 1.8136 | 1.3054 | 1.4318 |
| **Outer membrane protein OS=Klebsiella pneumoniae30660/NJST258_1 OX=1420012** | ***KPNJ1_00214*** | 2.0557 | 2.119 | 1.8903 |
| **Carbamoyl-phosphate synthase large chain OS=Klebsiella pneumoniaeOX=573** | ***carB_1*** | 2.1258 | 1.608 | 1.8181 |
| **Threonine--tRNA ligase OS=Klebsiella pneumoniae30660/NJST258_1 OX=1420012** | ***thrS*** | 2.5626 | 2.3453 | 2.1153 |
| **Bifunctional polymyxin resistance protein ArnA OS=Klebsiella pneumoniaeOX=573** | ***arnA*** | 2.6377 | 3.3296 | 2.9169 |
| **Phenylalanine--tRNA ligase beta subunit OS=Klebsiella pneumoniaesubsp. pneumoniae OX=72407** | ***pheT*** | 2.8273 | 1.8765 | 2.426 |
| **UDP-4-amino-4-deoxy-L-arabinose--oxoglutarate aminotransferase OS=Klebsiella pneumoniaesubsp. pneumoniae (strain HS11286) OX=1125630** | ***arnB*** | 2.9485 | 3.0798 | 2.7552 |
| **Valine--tRNA ligase OS=Klebsiella pneumoniaeOX=573** | ***valS*** | 3.0942 | 2.1397 | 2.6083 |

**S Table 4.-** Gene and their primer sequence details for Quantitative RT-PCR

| **Gene name** |  | **Primer Sequence** | **Tm** | **Length** |
| --- | --- | --- | --- | --- |
| *arnA* | F | 5' TCTCCTTCTACTATCGCAACC 3' | 50.9 | 143 bp |
| *arnA* | R | 5' TTCGCTTTCGCCATTGAC 3' | 50.8 |  |
| *arnB* | F | 5' TTATGATCGCCAGACCCAC 3' | 51.1 | 243 bp |
| *arnB* | R | 5' CGGATGATAAACAGATGCCAG 3' | 51.2 |  |
| *KPHS* | F | 5' GTGAAAACCGAGCAGAATCC 3' | 51.4 | 230 bp |
| *KPHS* | R | 5' TCTGAAAACGCCTTAATGCC 3' | 51 |  |

**S Figure 1.-** Volcano plots showing DEPs in different adapted *K. pneumoniae* In 1x MIC- 245 DEPs (105 upregulated and 140 downregulated), In 2x MIC - 383 (64 upregulated and 319 downregulated), and In 4x MIC - 175 (70 upregulated and 105 downregulated).

**S Figure 2.-** Core common DEPs expression profiling of 75 (15 upregulated and 60 downregulated) proteins.

**S Figure 3.-** Quantitative RT PCR gene expression profiling of *arnA*, *arnB* (upregulated) and *kphS* (downregulated)
